# Occupational class differences in pancreatic cancer survival: A population‐based cancer registry‐based study in Japan

**DOI:** 10.1002/cam4.2138

**Published:** 2019-04-05

**Authors:** Masayoshi Zaitsu, Yongjoo Kim, Hye‐Eun Lee, Takumi Takeuchi, Yasuki Kobayashi, Ichiro Kawachi

**Affiliations:** ^1^ Department of Public Health, Graduate School of Medicine The University of Tokyo Tokyo Japan; ^2^ Department of Social and Behavioral Sciences Harvard T.H. Chan School of Public Health Boston Massachusetts; ^3^ Korea Institute of Labor Safety and Health Seoul Republic of Korea; ^4^ Department of Urology Kanto Rosai Hospital Kawasaki Japan

**Keywords:** Japan, occupation, pancreatic cancer, population‐based, socioeconomic status, survival

## Abstract

**Background:**

Little is known about occupational class differences in pancreatic cancer survival.

**Methods:**

Using a population‐based cancer registry in Japan, 3 578 patients with incident pancreatic cancer (1970‐2011) were followed up for 5 years (median follow‐up time 0.42 years). We classified patients into four occupational classes based on their longest‐held jobs: white‐collar (professional and managers), service, blue‐collar, and those not actively employed. Using white‐collar class as the reference group, hazard ratios (HRs) and 95% confidence intervals (CIs) for overall death were estimated by Cox proportional hazard model. Covariates included age, sex, and year of diagnosis. Prognostic variables (pathology, stage, and treatment) and smoking behaviors were additionally adjusted as possible mediating factors.

**Results:**

Overall survival was poor in this population (median, 0.50 and 0.33 years in white‐collar and service classes, respectively). Compared with white‐collar patients, survival was significantly poorer across all occupational classes, most pronounced in the service worker group: mortality HRs ranged from 1.11 (95% CI 1.00‐1.24) in blue‐collar workers to 1.24 (95% CI 1.12‐1.37) in service workers. Even after controlling for potential mediating factors, service workers showed worse survival.

**Conclusion:**

We documented occupational class disparities in pancreatic cancer survival in Japan. Even in the setting of lethal prognostic cancer with universal health coverage, high‐occupational class groups may enjoy a health advantage.

## 1 INTRODUCTION

Pancreatic cancer accounts for ~4% of cancer‐related deaths worldwide, and the global burden of pancreatic cancer is increasing.^1^ Pancreatic cancer remains one of the most lethal types of cancer because effective screening program remains a significant challenge: that is, although prognostic improvement has been observed for early‐stage pancreatic cancer recently, most cases are still detected late.^2^, ^3^, ^4^, ^5^ For example, in 2016, the total number of deaths from pancreatic cancer in Japan was 33 475 (male 17 060 and female 16 415), and the 5‐year relative survival rates were 7.9% in men and 7.5% in women, respectively.^6^ The Japan Pancreatic Cancer Registry, administrated by the Japan Pancreas Society since 1981, suggests a favorable prognosis for early‐stage pancreatic cancer, with their 30‐year accumulated data (the median survivals for the Union for International Cancer Control [UICC]‐stage 0, Ia, and Ib were, respectively, 12.9, 10.0, and 8.4 years).^5^ By contrast, the prognosis for late‐stage cancer remains poor (the median survivals for UICC‐stage IIa, IIb, III, and IV were, respectively, 2.0, 1.3, 0.8, and 0.4 years), and most of the patients were detected at this late stage (87.9%, 20 745 of 23 609 patients).^5^

According to the theory of “SES as a fundamental cause of disease” advocated by Link and Phelan (1995), high‐socioeconomic status (SES) groups manage to enjoy a health advantage for a broad range of conditions regardless of their specific etiology.^7^ This health advantage is believed to extend to the survival of patients diagnosed with most forms of cancer, even though high‐SES individuals may be at higher risk of *incidence* of cancer at specific sites such as breast and prostate cancer.^8^, ^9^

Yet, studies that tried to examine socioeconomic disparities in pancreatic cancer survival remain scarce, particularly in non‐western contexts, and the results have not been consistent. For example, studies in the USA and the Netherlands suggest that pancreatic cancer patients from low‐SES backgrounds have worse prognosis compared with their high‐SES counterparts (a 7% to 16% difference in 5‐year overall survival).^10^, ^11^, ^12^ In the same studies, the socioeconomic disparity in pancreatic cancer survival was partially mediated by disparities in early diagnosis and access to surgical treatment,^10^, ^11^, ^12^ that is, two key determinants for improving pancreatic cancer prognosis.^3^, ^4^, ^5^, ^13^, ^14^ By contrast, one study from the Whitehall cohort in England did not show a significant association between occupational class and pancreatic cancer mortality (rate ratio of clerical workers vs professionals/executives 0.95, 95% CI 0.59‐1.51).^15^

Accordingly, the goal of the present study was to examine the association between occupational class and pancreatic cancer survival in Japan. Using a population‐based cancer registry dataset with pancreatic cancer, we sought to examine whether occupational class differences exist in pancreatic cancer survival in Japan and determine whether the disparity persists even after controlling for clinicopathological and treatment factors.

## 2 METHODS

### 2.1 Data setting

Using a large, population‐based dataset (1970‐2016) of Kanagawa Cancer Registry (KCR), we conducted a 5‐year survival analysis of pancreatic cancer. Details and accuracy of the study database have been previously described elsewhere.^16^, ^17^, ^18^, ^19^ Briefly, Kanagawa Prefecture, a metropolitan prefecture located close to Tokyo, has a population of over nine million, which is the second largest prefecture in Japan and covers approximately 7% of the Japanese population. KCR is one of the largest local cancer registries in Japan. Well‐trained tumor registrars certified by the training program of Japanese Association of Cancer Registries, whose program is accredited by the Surveillance, Epidemiology, and End Results program, are responsible for data collection. The data included basic information (sex, age, date of diagnosis, date of death/last follow‐up), and clinical information (pathology, stage, treatment).

Uniquely, KCR collected information on occupation and smoking behaviors if available during the study period (~10% of the registered cases). However, these data are no longer obtained due to the change of data management practice. KCR automatically updates dates of death/last follow‐up with population registers and death certificates, and previous diagnosis codes are updated to be consistent with changes in coding practice.

We obtained a de‐identified dataset under the research agreement between the authors and KCR, and the research ethics committees of The University of Tokyo, Tokyo (Protocol Number 3891‐4) approved the study.

### 2.2 Main outcome and study subjects

The main outcome was overall survival, defined by the observation duration (years) from the date of initial diagnosis to the date of death/last follow‐up.

From all 34 226 pancreatic cancer patients registered in the KCR who were diagnosed with incident pancreatic cancer (C25 in International Classification of Diseases, 10th revision) between 1970 and 2011, we excluded those who had missing data for occupational information (30 648 patients, 89.6%), leaving a retrospective cohort comprised with 3 578 study subjects who had complete occupational information for analysis.

### 2.3 Occupational class

From the longest‐held occupation listed in KCR based on Japan Standard Occupational Classification, we classified occupational class into four groups, as follows^8^, ^9^, ^20^: white‐collar class (eg, professional, engineering, administrative, and managerial workers), service class (eg, clerical, sales, and service workers), blue‐collar class (eg, agriculture, forestry, fishery, manufacturing, transport and machine operation, construction, mining, carrying, security, cleaning, packaging workers), and “others,” who were not actively employed in paid employment (eg, homemakers, students, and unemployed workers). Distribution of occupational class (Table 1) paralleled the Japanese national statistics, as well as the nationwide clinical and occupational data from the Inpatient Clinico‐Occupational Database of Rosai Hospital Group (ICOD‐R) in Japan.^8^, ^9^, ^20^

**Table 1:** Characteristics of 3 578 pancreatic cancer patients in Kanagawa Cancer Registry

Characteristics	Mean (SD) or number (%)^a^				
	All n = 3 578	White‐collar n = 655	Service n = 1 029	Blue‐collar n = 849	Not employed n = 1 045
Incidence rate, person‐year*	1.13	0.98	1.28	1.20	1.08
Median survival time, y*	0.42	0.50	0.33	0.33	0.42
Women*	705 (20%)	136 (21%)	346 (34%)	83 (9.8%)	140 (13%)
Age, y*	63 (11)	64 (12)	63 (11)	64 (11)	61 (11)
Year of diagnosis*	1994 (9)	1994 (9)	1994 (9)	1993 (9)	1996 (9)
Treatment	n = 3 398	n = 622	n = 988	n = 800	n = 988
Any surgery	1 382 (41%)	256 (41%)	390 (39%)	313 (39%)	423 (43%)
Any chemotherapy	1 509 (44%)	273 (44%)	420 (43%)	355 (44%)	461 (47%)
Stage	n = 365	n = 62	n = 83	n = 65	n = 155
Late stage	336 (92%)	56 (90%)	73 (88%)	64 (98%)	143 (92%)
Histological type	n = 1 867	n = 378	n = 519	n = 416	n = 554
PDCA	1 698 (91%)	333 (88%)	478 (92%)	380 (91%)	507 (92%)
Histological grade	n = 755	n = 141	n = 222	n = 165	n = 227
High grade	246 (33%)	44 (31%)	67 (30%)	60 (36%)	75 (33%)
Smoking habits	n = 669	n = 127	n = 164	n = 122	n = 256
Never	284 (42%)	64 (50%)	78 (48%)	46 (38%)	96 (38%)
Former	202 (30%)	29 (23%)	48 (29%)	38 (31%)	87 (34%)
Current	183 (27%)	34 (27%)	38 (23%)	38 (31%)	73 (29%)

PDAC, pancreatic adenocarcinoma.

a Percentage may not total 100 because of rounding.

*P‐values <0.05 for ANOVA, chi‐squared test, or logrank test across occupational classes.

### 2.4 Covariates

Covariates included sex, age, and year of diagnosis. Year of diagnosis was adjusted to control for potential secular changes such as occupational structure and treatment regimens. Additionally, based on the methodology used in previous studies,^10^, ^11^, ^12^, ^15^, ^17^, ^21^ clinicopathological variables were included in the regression analyses as potential mediating variables as follows: pancreatic adenocarcinoma (identified by International Classification of Disease for Oncology, Third edition pathological codes; 8050, 8140‐8147, 8160‐8162, 8180‐8221, 8250‐8507, 8514, 8520‐8551, 8560, 8570‐8574, 8576, 8940, 8941), pathological grade (grade 3 or 4 [high‐grade] vs grade 1 or 2 [low‐grade]), summary stage (regional invasion and distant metastasis [late stage] vs localized [early stage]), and any performed surgery (yes/no) or chemotherapy (yes/no), as well as smoking behaviors (never/former/current). As in previous studies with a population‐based cancer registry that follows the criteria of summary stating system in Surveillance, Epidemiology, and End Results,^11^ our staging criteria were different from the TNM staging guidelines because TNM staging was mostly not available through this study period in KCR.^17^

### 2.5 Statistical analysis

The 5‐year overall survivals were estimated, using the Kaplan‐Meier method. In our main analytic model (model 1), hazard ratios (HRs) and 95% confidence intervals (CIs) for overall death were estimated by Cox proportional hazard model, minimally adjusted for basic characteristics (sex, age, and year of diagnosis). The white‐collar class served as the reference group for all analyses. We adjusted for treatment (surgery and chemotherapy) in model 2 and additionally adjusted for stage in model 3 as potential mediating variables. Finally, in the full models, we adjusted for all potential mediating variables including treatment, stage, pathology, and smoking behaviors in model 4.

For sensitivity analysis, multiple imputation was applied for missing data. Except for basic characteristics (sex, age, year of diagnosis, and survival time), records were missing data on mediating factors: that is, 5.0% (180 patients) of treatment, 89.8% (3 213 patients) of stage, 69.4% (1 711 patients) of pathology, and 81.3% (2 909 patients) of smoking behaviors were missing. Excluding those with missing data may lead to biased inference; therefore, we conducted multiple imputation for missing data among the 3, 578 study subjects with all variables used for analysis, and 20 imputed datasets were generated (Table 1).^8^, ^9^, ^20^, ^22^ In a supplemental data analysis, due to the background and survival differences between those who completed occupational information and those who did not complete occupational information (Table S1 and Figure S1), we performed a regression analysis among all 34 226 pancreatic cancer patients (including 30 648 patients who did not have complete occupational information), with multiple imputation. In addition, we estimated HRs for the 1‐year overall survival.

Alpha was set at 0.05, and all *P*‐values were two sided. Data were analyzed using STATA/MP13.1 (StataCorp LP, College Station, TX).

## 3 RESULTS

**Figure 1:**
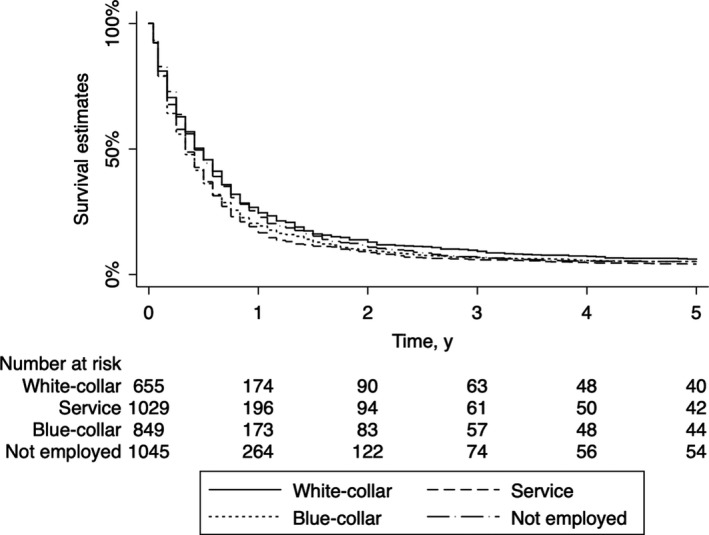
Kaplan‐Meier survival estimate curves for 5‐year overall survival in pancreatic cancer patients. Survival was estimated using the Kaplan‐Meier method in 3 578 patients, with right censoring at the 5‐year point. Logrank test: *P* < 0.001

The characteristics of the study patients are shown in Table 1. Overall, survival was poor in this population (median 0.42 years [5.0 months]). Between occupational classes, the distribution of baseline characteristics differed, while clinicopathological features and smoking behaviors did not differ (Table 1). The 5‐year overall survivals showed a slight occupational class difference (logrank test, *P* < 0.001, Figure 1): for example, the median survival time for white‐collar and service workers were, respectively, 0.50 years [6.0 months] and 0.33 years [4.0 months] (Table 1).

In the minimally adjusted Cox proportional hazard model (model 1), compared with white‐collar workers, survival was significantly poorer across all occupational classes, most pronounced in the service worker group (Table 2): HRs ranged from 1.11 (95% CI 1.0002‐1.24) in the blue‐collar class to 1.24 (95% CI 1.12‐1.37) in the service class. Occupational class differences were not attenuated and remained significant on adjustment of prognostic variables of treatment and stage (model 2 and model 3, Table 2). Even after maximally adjusting for clinicopathological factors and smoking behavior, the difference remained significant in the service class: HR 1.90 (95% CI 1.01‐3.57, model 4, Table 2).

**Table 2:** Hazard ratios and 95% confidence intervals for 5‐year overall survival estimated with Cox proportional hazard model

Characteristics	Hazard ratio (95% confidence interval)			
	Model 1 n = 3 578	Model 2 n = 3 398	Model 3 n = 356	Model 4 n = 140
Occupational class				
White‐collar	1.00	1.00	1.00	1.00
Service	1.24 (1.12, 1.37)***	1.22 (1.10, 1.35)***	1.51 (1.04, 2.18)*	1.90 (1.01, 3.57)*
Blue‐collar	1.11 (1.00, 1.24)*	1.10 (0.99, 1.22)	1.51 (1.02, 2.24)*	1.79 (0.90, 3.57)
Not employed	1.11 (1.01, 1.23)*	1.14 (1.03, 1.27)*	1.30 (0.94, 1.79)	1.45 (0.84, 2.52)
Women	0.90 (0.82, 0.98)*	0.94 (0.86, 1.03)	1.14 (0.89, 1.47)	1.20 (0.72, 2.01)
Age	1.01 (1.01, 1.01)***	1.01 (1.00, 1.01)***	1.00 (0.99, 1.01)	0.99 (0.97, 1.01)
Year of diagnosis	0.98 (0.98, 0.99)***	0.98 (0.97, 0.98)***	1.07 (1.00, 1.15)*	1.05 (0.91, 1.20)
Any surgery		0.51 (0.47, 0.55)***	0.35 (0.26, 0.47)***	0.18 (0.11, 0.29)***
Any chemotherapy		0.87 (0.81, 0.94)***	0.59 (0.44, 0.78)***	0.43 (0.25, 0.75)**
Late stage			2.88 (1.74, 4.78)***	1.53 (0.61, 3.80)
PDAC				0.80 (0.32, 1.99)
High grade				2.36 (1.60, 3.48)***
Smoking habits				
Never				1.00
Former				1.19 (0.70, 2.01)
Current				1.10 (0.64, 1.88)

PDAC, pancreatic adenocarcinoma.

*P < 0.05.

**P < 0.01.

***P < 0.001.

In sensitivity analyses with multiply imputed data, the same pattern of the main results was observed (Table 3): the fully adjusted HRs for service workers and those who were not actively employed were, respectively, 1.37 (95% CI 1.10‐1.73) and 1.30 (95% CI 1.03‐1.64, model 4). In the supplemental data analysis among all 34 226 pancreatic cancer patients in the KCR, the pattern of occupational class disparities was almost identical (Table S2). Additionally, the result estimated for the 1‐year overall survival was almost identical (eg, HR in the service class, 1.28; 95% CI 1.15‐1.43, model 1).

**Table 3:** Results of Cox proportional hazard model with multiple imputation among 3 578 pancreatic cancer patients who had complete occupational information

Characteristics	Hazard ratio (95% confidence interval)			
	Model 1 n = 3 578	Model 2^a^ n = 3 578	Model 3^a^ n = 3 578	Model 4^a^ n = 3 578
Occupational class				
White‐collar	1.00	1.00	1.00	1.00
Service	1.24 (1.12, 1.37)***	1.21 (1.10, 1.35)***	1.23 (1.11, 1.37)***	1.37 (1.10, 1.73)**
Blue‐collar	1.11 (1.00, 1.24)*	1.10 (0.99, 1.23)	1.09 (0.98, 1.22)	1.20 (0.94, 1.53)
Not employed	1.11 (1.01, 1.23)*	1.14 (1.03, 1.26)*	1.13 (1.02, 1.26)*	1.30 (1.03, 1.64)*
Women	0.90 (0.82, 0.98)*	0.93 (0.85, 1.02)	0.93 (0.85, 1.02)	0.78 (0.62, 0.96)*
Age	1.01 (1.01, 1.01)***	1.01 (1.00, 1.01)***	1.01 (1.00, 1.01)***	1.00 (1.00, 1.01)
Year of diagnosis	0.98 (0.98, 0.99)***	0.98 (0.98, 0.98)***	0.98 (0.98, 0.99)***	0.97 (0.96, 0.98)***
Any surgery		0.52 (0.48, 0.56)***	0.53 (0.49, 0.58)***	0.43 (0.36, 0.52)***
Any chemotherapy		0.88 (0.82, 0.94)***	0.83 (0.77, 0.90)***	0.76 (0.65, 0.90)**
Late stage			4.49 (1.59, 12.6)**	3.05 (1.28, 7.07)*
PDAC				1.16 (0.77, 1.74)
High grade				1.52 (1.29, 1.79)***
Smoking habits				
Never				1.00
Former				0.95 (0.70, 1.28)
Current				0.93 (0.68, 1.27)

PDAC, pancreatic adenocarcinoma.

a Data for model 2, 3, and 4 were estimated with 20 imputed datasets.

*P < 0.05.

**P < 0.01.

***P < 0.001.

## 4 DISCUSSION

As far as we are aware, this is the first report of occupational class differences in pancreatic cancer survival in Japan, where universal health coverage (established in 1961) has minimized socioeconomic disparities in cancer mortality by providing equal access to standard care and treatment, irrespective of SES.^23^ Compared with patients working in managerial/professional positions, we observed 11%‐24% poorer survival in those working in service and blue‐collar class positions. Previous studies implied that poorer pancreatic cancer survival in socioeconomically disadvantaged individuals stemmed from lower access to treatment (especially surgical treatment)^10^, ^11^, ^12^ and potential early diagnosis/timely treatment.^24^, ^25^ However, in this study, occupational class difference in pancreatic cancer survival was not attenuated and remained statistically significant even after controlling for potential mediating factors, suggesting that occupational class can be considered as a determinant of pancreatic cancer prognosis, comparable in clinical significance as conventional prognostic factors.

The observed occupational class difference in pancreatic cancer survival may be partly explained by differences in exposure to chronic stress, affecting biological/psychological pathways, in combination with job stress among low‐occupational class individuals. For example, chronic psychological stress is known to increase pancreatic cancer growth and invasion via multiple neurotransmitter pathways, including *β*‐adrenergic stimulating and suppression of the inhibitory *γ*‐aminobutyric acid (GABA) in basic experiments.^26^, ^27^, ^28^ Compared with non‐stressed mice, stress‐exposed mice showed an increase in the progression rate of pancreatic cancer volume by 10.9% per day^27^ and a decrease in the median survival by 13.5%.^28^ Additionally, *β*‐blocker administration or GABA replacement prevented the effects of chronic stress on pancreatic cancer growth.^26^, ^27^, ^28^ In previous epidemiological studies, occupational class was associated with job stress,^29^, ^30^ as well as other biological stress markers, such as C‐reactive protein.^31^, ^32^ However, the association between job stress and pancreatic cancer risk remains unclear. Therefore, future studies in this area are needed to better understand the conditions under which these factors might contribute to pancreatic cancer prognosis.

The fundamental cause theory may explain the observed occupational class difference.^7^ According to this theory, we could assume that no matter how universal health coverage covers comprehensive cancer treatments irrespective of SES, the high‐occupational class group may still manage to enjoy an advantage to improve their prognosis. In this study, the socioeconomic gap of healthcare access, which was a significant mediator in previous studies in the USA and the Netherlands,^10^, ^11^, ^12^ might not play a critical role in our study because of universal health coverage.^23^ Another behavioral mechanism, such as early diagnosis/timely treatment, might play a role, aligned with fundamental cause theory. It is because compared with low‐SES counterparts, high‐SES patients are likely to have an early diagnosis/timely treatment,^24^, ^25^ which is the key factor in determining prognosis in such a very aggressive and lethal cancer.^3^, ^4^, ^5^, ^13^, ^14^

Several limitations should be noted. First, although our dataset was based on a population‐based cancer registry, complete data were limited for occupational class and prognostic variables due to the majority of missing data, thereby limiting external generalizability. Additionally, we were not able to fully assess possible mediating pathways through prognostic parameters (eg, stage) due to a large amount of missing data. However, our sensitivity analysis with multiple imputation yielded almost identical results. Additionally, the distribution of occupational class in this local population‐based data paralleled the Japanese national statistics and the nationwide clinical and occupational data from ICOD‐R^8^, ^9^, ^20^ In addition, the distribution of stage and survival rates were comparable to previous data from the Japan Pancreatic Cancer Registry.^5^ Second, we could not assess the contribution of other relevant socioeconomic factors (ie, educational attainment and income) on pancreatic cancer survival due to the limitation of data.^33^ We also could not assess other possible mediating variables such as alcohol consumption, physical activity, metabolic disorders (eg, diabetes), and environmental exposure to chemical compounds.^34^ However, most of these possible mediating factors were not associated with pancreatic cancer mortality in the Whitehall study.^15^ Finally, our analyzed mediating variables were less detailed (eg, binary categories of pathology, stage and treatment) compared with previous studies using detailed classification.^10^, ^11^, ^12^, ^21^ Even though we adjusted for clinical stage, microenvironmental progression of the disease by delayed diagnosis/treatment—which may be potentially associated with occupational class—could explain residual differences in prognosis. However, the direction and magnitude of socioeconomic disparities were similar between previous studies (7%‐16% poorer in survivals in low‐SES patients) and our result (11%‐24% poorer in survivals in low‐SES patients).^10^, ^11^, ^12^, ^21^

Despite these limitations, the strengths of our study included a relatively large sample size, one of the largest studies conducted for evaluating the association between occupational class and pancreatic cancer survival in the non‐western setting. In addition, although previous studies used a regional‐level (but not individual‐level) socioeconomic indicator^10^, ^11^, ^12^, ^21^ and the Japanese national cancer registry has not collected relevant socioeconomic indicators (ie, occupation, educational attainment, or incomes), our study with the population‐based KCR directly measured an individual‐level socioeconomic indicator of the longest‐held occupation, which may introduce less misclassification compared to other alternative occupational indicators (eg, current or most recent jobs).^8^, ^9^, ^20^, ^35^ Further studies should address the occupational class differences in pancreatic cancer survival, trying to use data linkage approach between cancer registries and relevant population‐based data with socioeconomic indicators (such being seen in Nordic countries).^36^, ^37^ Health service research approach, using linkage data of relevant occupational/health insurance data and national death registries (such being seen in South Korea), may also be promising.^38^, ^39^

In conclusion, we observed occupational class differences in pancreatic cancer survival in Japan. Even in the setting of lethal prognostic cancer with universal health coverage, high‐occupational class groups may enjoy a health advantage. Nevertheless, specific reasons and biological pathways to explain our observed occupational class disparities remain unclear. Hence, future studies should attempt to integrate all potential pathways from molecular pathology to clinical/social epidemiology.

## CONFLICT OF INTEREST

The authors declare no potential conflicts of interest.

## AUTHOR CONTRIBUTIONS

Conception and design: M Zaitsu, I Kawachi. Development of methodology: M Zaitsu, Y Kim, I Kawachi. Acquisition of data, analysis and interpretation of data: M Zaitsu. Writing, review and/or revision of the manuscript: M Zaitsu, Y Kim, HE Lee, T Takeuchi, Y Kobayashi, I Kawachi. Administrative, technical, or material support: M Zaitsu, T Takeuchi, Y Kobayashi, I Kawachi. Study supervision: M Zaitsu, Y Kobayashi, I Kawachi.
