# Supplementary material for: Occupational class differences in pancreatic cancer survival: A population‐based cancer registry‐based study in Japan
Source: Cancer Med. 2019 Apr 5;8(6):3261–8. doi: 10.1002/cam4.2138 (PMC6558482; doi:10.1002/cam4.2138)
Supplement: Supplementary file 1 [file CAM4-8-3261-s001.docx]

**Figure S1. Kaplan–Meier survival estimate curves for 5-year overall survival for 3,578 pancreatic cancer patients who completed occupational information and 30,648 pancreatic cancer patients who did not complete occupational information.** Logrank test: P <.001.

**
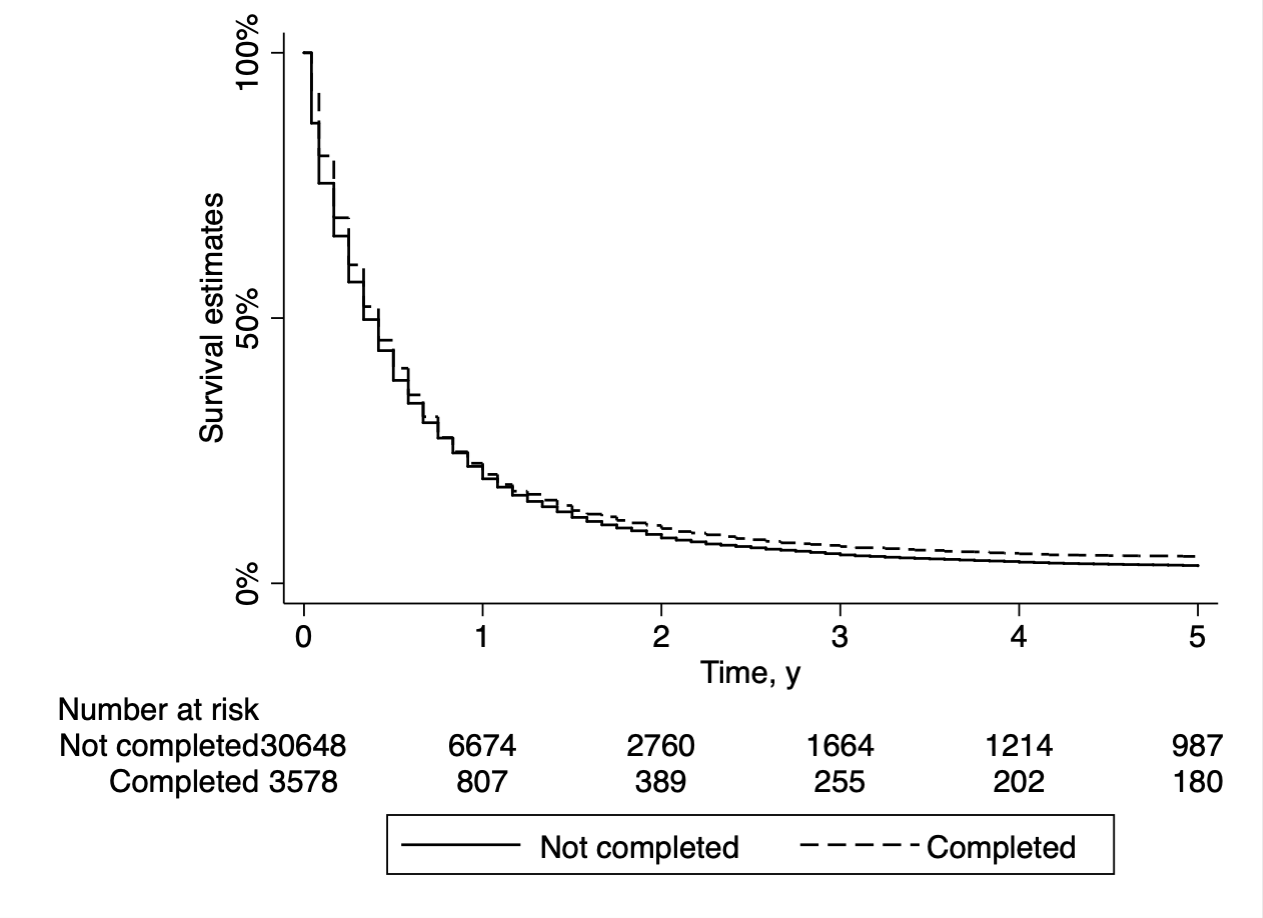
**

**Table S1. Differences between those who completed occupational information and those who did not complete occupational information**

| Characteristics | Not completed  n = 30,648 | Completed  n = 3,578 | *P*-value^a^ |
| --- | --- | --- | --- |
| Incidence rate for overall death, person-year | 1.28 | 1.14 | <.001 |
| Median survival time, y | 0.33 | 0.42 | <.001 |
| Women | 14,189 (46.3%) | 705 (19.7%) | <.001 |
| Age, y | 70 (12) | 63 (11) | <.001 |
| Year of diagnosis | 1998 (11) | 1994 (9) | <.001 |

^a^ *P*-values: logrank test, t-test, and chi-squared test.

**Table S2. Results of Cox proportional hazard model with multiple imputation among 34,226 pancreatic cancer patients who had complete occupational information**

| Characteristics | Hazard ratio (95% confidence interval)^a^ | | | |
| --- | --- | --- | --- | --- |
|  | Model 1 | Model 2 | Model 3 | Model 4 |
| Occupational class |  |  |  |  |
| White-collar | 1.00 | 1.00 | 1.00 | 1.00 |
| Service | 1.10 (1.04, 1.16)** | 1.08 (1.03, 1.13)** | 1.08 (1.03, 1.13)** | 1.16 (0.97, 1.38) |
| Blue-collar | 1.15 (0.97, 1.12) | 1.03 (0.97, 1.10) | 1.02 (0.94, 1.11) | 1.08 (0.91, 1.29) |
| Not employed | 1.07 (1.01, 1.13)* | 1.08 (1.02, 1.14)** | 1.08 (1.003, 1.16)* | 1.14 (0.94, 1.39) |
|  |  |  |  |  |
| Women | 0.94 (0.92, 0.97)*** | 0.95 (0.92, 0.97)*** | 0.95 (0.92, 0.98)** | 0.96 (0.87, 1.06) |
| Age | 1.02 (1.02, 1.02)*** | 1.01 (1.01, 1.01)*** | 1.01 (1.01, 1.02)*** | 1.01 (1.01, 1.02)*** |
| Year of diagnosis | 0.99 (0.98, 0.99)*** | 0.98 (0.98, 0.99)*** | 0.99 (0.98, 0.99)*** | 0.97 (0.96, 0.97)*** |
| Any surgery |  | 0.63 (0.62, 0.65)*** | 0.65 (0.63, 0.67)*** | 0.44 (0.40, 0.48)*** |
| Any chemotherapy |  | 0.89 (0.87, 0.92)*** | 0.85 (0.82, 0.88)*** | 0.78 (0.72, 0.85)*** |
| Late stage |  |  | 2.38 (1.89, 2.99)*** | 2.24 (1.72, 2.93)*** |
| PDAC |  |  |  | 1.23 (0.999, 1.53) |
| High grade |  |  |  | 1.43 (1.32, 1.55)*** |
| Smoking habits |  |  |  |  |
| Never |  |  |  | 1.00 |
| Former |  |  |  | 1.06 (0.92, 1.23) |
| Current |  |  |  | 1.09 (0.94, 1.26) |

Abbreviation: PDAC, pancreatic adenocarcinoma. * *P* <.05. ** *P* <.01. *** *P* <.001.

^a^ Data were estimated with 20 imputed datasets. The numbers of missing data were, respectively, as follows: occupational class (n=30,648, 90%), surgery (n=14,109, 41%), chemotherapy (n=14,109, 41%), stage (n=29,399, 86%), pathological type (n=25,776, 75%), pathological grade (n=30,772, 90%), and smoking habits (n=30,519, 89%).
